# Embedding and Integrating a Digital Patient Management Platform Into Everyday Primary Care Routines: Qualitative Case Study

**DOI:** 10.2196/30527

**Published:** 2022-02-22

**Authors:** Susanne Frennert, Gudbjörg Erlingsdóttir, Mirella Muhic, Christofer Rydenfält, Veronica Milos Nymberg, Björn Ekman

**Affiliations:** 1 Department of Design Sciences Lund University Lund Sweden; 2 Department of Informatics Umeå University Umeå Sweden; 3 Department of Clinical Sciences, Malmö Lund University Malmö Sweden

**Keywords:** digital patient management platform, primary care, normalization process theory coherence, cognitive participation, collective action, reflexive monitoring

## Abstract

**Background:**

Traditional primary care is characterized by patient consultations via phone and physical visits. However, the current development in Swedish primary care is to blend digital solutions with traditional solutions. This paper addresses this development by examining the normalization of embedding and integrating a digital health care platform into everyday care routines in a primary care clinic. The digital health care platform enables both synchronous (video calls) and asynchronous (chat) communication, as well as self-registration of patient data using automated questions and forms requiring the patient’s input.

**Objective:**

This study aims to explore the work that health care professionals (HCPs) have to undertake to implement and sustain a digital health care platform as part of their everyday work practice.

**Methods:**

HCPs were observed and interviewed to assess their individual and collective engagement and the mechanisms involved in the implementation of the digital platform and its effects on everyday work routines. The normalization process theory (NPT) was used to frame the data analysis.

**Results:**

The analysis identified several themes related to the four NPT constructs: coherence, cognitive participation, collective action, and reflexive monitoring. The use of these constructs enabled the analysis to identify ways of supporting implementation. For example, it showed the benefits of having implementation champions and scheduling work hours for HCPs to use the platform. The analysis also revealed a theme of *materiality* that deviated from the NPT constructs, as NPT gives ontological priority to human actors and social structures.

**Conclusions:**

Digital health care platform implementation is a complex process. Our findings provide insights into how individual and collective actions can be supported to embed and integrate a digital platform into everyday care routines. Primary health care organizations need to involve HCPs throughout the implementation process by reorganizing work and providing frequent feedback loops. HCPs are more likely to engage with and commit to changing practices if they perceive the digital platform to be beneficial compared with the current practice. However, they also need resources (eg, time, training, and continuous support) to put the platform into practice. Patient engagement and appraisal are important elements in implementation. Unless patients are willing to use the platform, there is no motivation for HCPs to embed the digital platform into everyday care practice.

## Introduction

### Background

Sweden, similar to many other countries, is undergoing demographic changes, resulting in an increasing older population and a shortage of health care professionals (HCPs). Thus, the Swedish health care system is in transition, aiming to move from reactive to proactive care, with a strong focus on preventive care that is aimed at keeping people out of hospitals. The change from inpatient care to outpatient care means that the locus of care is increasingly shifting toward primary care and patients’ homes.

Enhanced digitalization in primary care is seen as a way of meeting current and future challenges [1,2]. The digital transformation of health care is associated with the promise of an improved public sector, with self-care and greater self-management of the health of individuals, resulting in increased cost efficiency. Digitalization in primary care entails converting parts of the care process from analog to digital to meet the needs of citizens, patients, decision-makers, HCPs, and clinics. In Sweden, the digital transformation of primary care has involved several different kinds of digital solutions, including web-based physicians (general practitioners performing digital consultations) [3], self-monitoring mobile apps [4], patient access to their electronic medical records (OpenNotes) [5], digital reminders for medication [6], and digital platforms for patient management [7,8]. This paper concerns the normalization process of a digital platform for patient management. The platform, which will be referred to as Wolf in this paper, was developed by a Swedish company and is procured and used by both private and publicly run primary health care centers and other primary care operations. In Sweden, all types of primary care are tax funded. Both public and private primary care providers enjoy a large degree of economic autonomy. This means that capital investment decisions, such as the one studied here, are made by the owners of the primary care providers, who also finance the investments. Such investments have no direct effect on user fees and are made in the hopes of providing more efficient and less costly services.

The same digital platforms as studied in this paper have been studied in different primary care settings [7-9]. However, the findings show diverse results and have not explicitly focused on the normalization process. For example, Entezarjou et al [9] investigated how family medicine physicians and nurses at primary care centers experienced the implementation and use of digital communication in the form of automated patient interviewing software and chat-based patient–provider communication. Their findings indicated that the use of the digital platform streamlined consultations with patients and improved interdisciplinary communication. The study also found that digitalization was perceived as essential because of the expectations of patients and HCPs. However, their interviewees acknowledged that digital communication did not suit all patients and all kinds of patient requests, and thus, they perceived the platform as only a partial solution [9]. Cajander et al [7] studied a digital platform, which they called a digitalized and automated patient-centric service, in their research on how it affected work engagement among nurses in a national telephone nursing service. They found that nurses felt stressed by having to deal with many patients in parallel, knowing that patients were waiting in the queue to chat with them. As the chat function offered asynchronous communication, the nurses spent time waiting for patients to respond and checking over and over to see whether any patients in parallel, ongoing conversations had responded while, at the same time, a queue of new patients was piling up. Another disadvantage highlighted by the nurses was the lack of feedback from physicians and patients. This finding contrasts with the findings of Entezarjou et al [9], who indicated that the digital platform increased interdisciplinary communication and streamlined patient consultations. However, it is not clear whether the lack of feedback was because of the use of the digital platform or whether this was also common in the ordinary care routine before the implementation of the platform [7]. Another study [8] of the same platform, conducted at primary care centers, indicated that written communication with patients was perceived as more time consuming than communication by phone. It also produced large amounts of text, which made it more difficult to quickly scan information to get an overview of patient concerns. These findings are also somewhat at odds with the finding of Cajander et al [7] that nurses felt that written communication with patients was less emotionally demanding. The prime advantage of using the digital platform, according to Eldh et al [8], was the possibility for patients to upload photos, which reduced physical patient visits. Their findings indicated that the system of automated triage facilitated response and consultation with patients. They conclude that the HCPs in the study lacked the resources needed to benefit from the platform and settled upon different routines themselves, with varied results [8].

The findings of the aforementioned studies indicate that several intertwined factors rather than just the digital platform per se affect the outcome in care practice. Thus, technology adaption and implementation in primary care are not straightforward; rather, it is a complex process that is affected by multiple factors (technological, social, structural, historical, economic, and political), different actors (HCPs, patients, next-of-kin, management, and politicians), organizations, ecologies of technologies, and core ideas (eg, patient safety, patient access, and efficiency). These are interrelated and afford certain kinds of care and working conditions while restraining others [10-12]. Past research shows that the implementation of this type of digital platform is a complex process involving a wide range of actors who translate means, actions, and objectives into care practices in different ways [13] and do not always render the expected effects [7]. Furthermore, early research on digital implementation in health care has been accused of being rich in data but *information poor* [10]. However, several theoretical tools for comprehending and illuminating implementation failures or successes have been developed [10,13-16]. One such explanatory framework is the normalization process theory (NPT) [15]. NPT identifies and explains the important mechanisms that promote or inhibit the implementation process. It allows for a systematic exploration of how and why (or not) a digital health care platform becomes normalized and sustained in health care practice. NPT “characterizes and explains implementation processes as interactions between ‘emergent expressions of agency’ (i.e. the things that people do to make something happen, and the ways they work with different components of a complex intervention to do so); and as ‘dynamic elements of context’ (the social-structural and social-cognitive resources that people draw on to realize that agency)” [17]. Other theoretical frameworks that have been used to better understand the implementation processes concerned with digital technologies in health care are the Rogers [18,19] diffusion of innovations theory and the technology acceptance model [20,21]. Although the latter is primarily concerned with the characteristics at the individual level that affect individuals’ intentions to use the technology, the former attempts to connect the macro level to the micro level or context of use. However, although the Rogers [18,19] diffusion of innovation theory considers the context, it does not account for the dynamics of the context in the way that NPT does.

### Aims and Objectives

This paper adds to the growing body of research on digital platforms in primary care and the understanding of how to facilitate implementation. We seek to explore whether and how a digital platform becomes part of the everyday primary care practice and the mechanisms involved in its implementation. NPT pays attention to what work people do, or need to do, for complex interventions to become a normal part of everyday work [15,16]. Therefore, in this paper, we explore whether and how the constructs of NPT are visible in the implementation of a digital platform in Swedish primary care and whether the use of NPT offers additional insights into more inductive approaches.

## Methods

### Overview

This study is part of a large project on the impacts of digitalization on the working environment of primary HCPs when transforming traditional primary care practices into combined digital and physical care practices. As part of the project, this qualitative study explores, through interviews and observations, the implementation process of a digital patient management platform in regard to NPT [15].

### Theoretical Framework: NPT

NPT [15,17] is a middle-range theory that goes beyond identifying barriers to and facilitators of a specific implementation and focuses on the means by which people, individually and together, act and try to configure an implementation process. NPT describes four constructs that shape an implementation process: *coherence*, which is the extent to which an innovation is understood as meaningful, desirable, and practicable; *cognitive participation*, which is the enrollment of key individuals to drive the implementation of the innovation; *collective action*, which is the work that operationalizes the innovation into practice; and *reflexive monitoring*, which is the ongoing process of adjusting and appraising the innovation to sustain routines [17].

Our motive for using NPT is that the theory offers a way of understanding how HCPs individually and mutually engage in or foresee engagement with a digital platform in primary care and how specific organizational and social norms and values are being invented or reinvented in the interactions with the digital platform and other people [17,22,23]. As such, it allows for the anticipation of and reflection on the implications of technology implementation in practice to ensure informed implementation, use, and the governance of digital primary care solutions.

### The Setting

The provision of health care in Sweden is the responsibility of the 21 autonomous health care regions, the largest of which are the region of Stockholm, region of Västra Götaland (including the city of Gothenburg), and region of Skåne (including the city of Malmö). The regions ensure the universal provision of health care to the citizens of the regions through the direct ownership of clinics and hospitals or by means of contracting with private providers. Most hospitals are publicly owned by the regions, whereas approximately 40% of primary care providers are private. Most private primary care providers are owned by larger companies that run chains of primary care clinics.

In 2019, Sweden spent approximately 11% of the Gross Domestic Product on health care (compared with the Organization for Economic Cooperation and Development average of 8%). Health care is funded primarily by means of regional income taxes (approximately 82% of total health care expenditure). Approximately 15% of the overall spending is in the form of out-of-pocket expenditures, largely because of user fees for health care and cost sharing with respect to pharmaceuticals. Only a small, albeit increasing, portion of the total health care spending goes to private health insurance.

Our study focuses on HCPs employed at Albra, a primary health care center in Sweden (the health care center, the digital platform, the company, and interviewees are anonymized. The names Albra, Wolf, and company D are thus fictitious). It has approximately 40 employees representing different health care professions. These physicians, nurses, psychologists, rehabilitation coordinators, and health care administrators provide care to approximately 10,000 patients, which makes Albra an average-sized Swedish primary care center. Albra has a team-based approach with close collaboration between different HCPs. Similar to many other primary care centers in Sweden, Albra uses a nurse-based triage of patient errands. This means that the first point of contact is a nurse, who, during the telephone conversation, assesses whether the patient needs to see a physician or another HCP in the team. Primary care nurses in Sweden are both registered nurses and nurses with specialist competence in primary care (district nurses). District nurses can prescribe some medications, such as topical steroids and antihistamines, usually after an in-person assessment. In addition to a varying number of remote patient contacts (usually by phone) and other patient-related administrative tasks, physicians usually meet between 10 and 20 patients daily, with visits varying between 15 and 45 minutes depending on the type of visit (emergency or planned follow-up of chronic diseases). These patients are often triaged by the first-contact nurses before the booking. Primary and secondary care use different electronic medical records, and patients have limited access to medical information from their records by accessing a national platform called 1177 with a bank ID.

Albra was struggling with long patient queues and a stressful work environment, especially for nurses who were answering patients’ phone calls. Therefore, in December 2019, the management and staff decided to implement a digital health care platform known as Wolf, in an attempt to address these problems. The purpose of the implementation was to increase patient accessibility, improve resource use, and decrease the workload for HCPs (primarily nurses).

### The Platform

The digital platform, Wolf, was developed in Sweden in 2016 by people with backgrounds in health care to support work and patient management in primary and outpatient care. The platform has been widely implemented by private and public primary care centers in Sweden. It is also used in Norway, the Czech Republic, Poland, and England.

The purpose of the platform is to improve medical quality, resource use, and patient experience. In contrast to traditional Swedish primary care routines, the platform enables digital patient contact. Through the platform, patients can initiate contact with the health care center digitally instead of through phone calls. First, patients enter their symptoms and receive automated questions depending on their responses. The patient data are compiled as a medical report and communicated to the health care center via the platform. At the health care center, a nurse enters the platform, reads the medical report, and allocates the patient matter to appropriate health care personnel for attendance. Thereafter, communication and patient meetings take place either synchronously or asynchronously in the form of digital (video or chat) or physical meetings with different categories of HCPs.

### Data Collection

The collected data covered the initial phase of implementation and its outcomes. The data were collected using the following settings:

- Two half-day observations during the HCP training sessions with the digital platform provider
- Observation at a formal workplace meeting in which the HCPs could comment and reflect on the platform together with colleagues and management
- Qualitative interviews with HCPs (N=11)
- A qualitative interview with one of the main initiators and developers of the platform

All 3 observations were conducted by a researcher who sat in the 2 training sessions and the workplace meeting. The observed personnel were aware of the researcher’s identity and the reason for her presence. Notes were taken during all 3 observations. The notes were rewritten and added to the analysis.

The interviewees were recruited and interviewed by one of the authors. Data collection took place between January 2020 and June 2020. Each participant was interviewed individually by phone or video call because of the current COVID-19 pandemic. The interviews averaged 40 minutes and were digitally recorded and transcribed in full for in-depth analysis. The interview questions were directed at eliciting the views and experiences of Wolf and its implementation. They were asked to describe the implementation process and their role in it, how they learned about the digital platform and how to use it, their expectations about how the platform would affect their everyday practice, and how they then experienced the digital platform and its effect on their work.

### Analysis

Our analytical emphasis was on the meanings our interviewees gave to the digital platform, its implementation, and its perceived effect on their work. The constructs of NPT (ie, coherence, collective participation, collective action, and reflexive monitoring) served as a framework for identifying the work and meanings that the HCPs associated with the embedding and integration of the digital platform into everyday care practice [15]. According to NPT, an innovation is likely to become successfully integrated if (1) those affected by it and its implementation understand and agree on its adaption and usefulness in practice (*coherence*), (2) those affected by it and its implementation engage and commit to using the new innovation (*cognitive participation*), (3) operational work is done to enact the innovation as part of the practice (*collective action*), and (4) those affected by it and its implementation positively and continuously appraise the utility and usefulness of the platform (*reflexive monitoring*) [15,17].

We reviewed all transcripts and highlighted all texts that appeared to describe important issues related to the platform and its implementation. All highlighted text was then coded using an inductive approach [24] to identify themes. MM, GE, and SF each coded the material independently, and the identified themes were then discussed and scrutinized until consensus was reached. Fifteen themes were identified: *beneficial for patients*, *assistance for nurses*, *as a supplement*, *close collaboration*, *implementation champions*, *patient engagement*, *familiarity*, *parallel work practices*, *new communication patterns*, *ease of use*, *enhancing* *teamwork*, *relief and less distress*, *flexibility*, *workarounds,* and *materiality*.

After the coding, data for each theme were examined to determine whether the categories of NPT described by May et al [15,17] were present.

Themes that could not be coded into one of these NPT categories were coded under another label that captured the essence of individual and collective actions and the mechanisms involved in the implementation. We compared the extent to which the data were supportive of the constructs described by the NPT versus how much they represented different mechanisms involved in the implementation of the digital platform.

### Ethics

According to the Swedish Ethical Review Act (SFS 2003:460) [25], research that involves retrieval and handling of sensitive personal data or is likely to cause physical and psychological impact or in other ways harm the subjects is required to undergo ethical review. Data regarding participants' race, ethnic origin, political opinion, religious conviction and the like, is considered as having a sensitive character.

In the study presented in this article, no ethical approval was required according to the Swedish Ethical Review Act (SFS 2003:460), as we did not ask interviewees about their own health or other sensitive topics (see above what is considered as sensitive data).

Participation was optional, and all participants provided written consent. The participants were informed that they could withdraw at any time, for any reason, or for no reason at all. To guarantee anonymity, no names or places are mentioned in the text. The researchers followed the guidelines on research ethics issued by the Swedish Research Council [26].

## Results

### Overview

The group of 11 interviewees comprised 1 of the main initiators and developers of the platform, 4 nurses, 3 physicians, 2 managers, 1 psychologist, and 1 rehabilitation coordinator from the health care center. All participants were female, except for 2 of the physicians and the developer. They all provided rich and often highly reflexive accounts of their experiences of, and views about, the digital platform and its implementation. To unpack these issues, we present their experiences and views through the four constructs (coherence, cognitive participation, collective action, and reflexive monitoring) described by May et al [15,17]. We also present the theme of *materiality*, which deviates from NPT constructs.

### Coherence: Sense-Making Work HCPs Do When Implementing the Digital Platform

The first construct in NPT is coherence. Coherence or sense-making work refers to the process by which people give meaning and value to a new practice instigated by the utility of new technology and its implementation [15]. The sense-making process starts as soon as the ideas of a new implementation emerge. Thus, people not only make sense of an implementation based on their experience of it but also draw on promises, hopes, worries, and fears in regard to the new technology [27,28]. As a result, before the implementation starts, people already have ideas about what the technology is and how and whether it fits into their worldview, habits, and work. These ideas are not fixed and stable but are part of an iterative and collective sense-making process. The analysis revealed that the interviewees had a shared understanding of the aim of implementing the digital platform. Important coherence-related themes that emerged from the interview data were *beneficial for patients*, *assistance for nurses,* and *as a supplement* (Table 1).

**Table 1:** Overview: themes that adhered to the normalization process theory (NPT) constructs and the theme that deviated from the NPT.

NPT constructs and subthemes			Excerpts
**Coherence**			
	Beneficial for patients	“That it becomes easily accessible for the patients, that they can contact us when it isn’t...when we aren’t open, but rather when it suits them...that it is a way for them to reach us” [rehab coordinator 1]	
	Assistance for nurses	“First, to make the nurses’ situation easier—it is onerous for them to sit long days on the phone dealing with a lot of tasks. So it was about giving them good tools for triage and more variety in their work team.” [physician 3]	
	As a supplement	“...the idea is that we should transfer as many tasks as possible, and those tasks that are appropriate...We would like to shift as many as possible over to Wolf, but not everyone can use digital contacts, not everyone has that option, for various reasons. So the telephone will always be with us, that’s the way it is.” [operations area manager]	
**Cognitive participation**			
	Close collaboration	“I think that the staff from Wolf who come to us, it is very...it feels very positive. They are deeply engaged and able to answer questions and so on.” [psychologist 1]	
	Implementation champions	“We have had a physician here who has been positively disposed and sort of pulled us along. And I think that it has been much easier to get underway, given that it has actually been one of the physicians that has been the driving force.” [nurse 1]	
	Patient engagement	“I think it works really well. It’s actually been a positive surprise; I thought it would be much more difficult. But I think it has worked well. And the patients, sometimes they have doubts, but once they’re used it, they feel very comfortable with it, and often come back to that approach.” [nurse 3]	
	Familiarity	“I think it is very easy and very good. There are...the colours that are present are clear and good. And the dialogue boxes and how you should...one needs...It is easy to understand, it is easy to immerse yourself in it, so I think it’s great.” [nurse 2]	
**Collective action**			
	Parallel work practices	“You have to set aside time for it. It takes time. As long as its entered in our time sheets, which we make sure to do, then it goes well...Yes, but it has gotten more enjoyable in that it’s a change from sitting talking on the phone.” [nurse 4]	
	New communication patterns	“There are fewer disturbances for both nurses and physicians, given that asking advice from a colleague involves a push of a button rather than having to run up two flights of stairs and knock on a door, and then they would be busy and you’d have to do it again later. And a ton of similar bother.” [physician 3]	
**Reflexive monitoring**			
	Ease of use	“Over the years I’ve been working, there have been good and bad digital systems, so to speak, and they don’t always make things easier, but I think that Wolf is one of the systems that actually facilitates the work.” [physician 1]	
	Enhancing teamwork	“Yes, but a little more collaboration, I would have to add. We collaborated very well among ourselves before as well, but it is a bit easier when you can write in the platform and you don’t have to hunt for one another physically” [physician 2]	
	Relief and less distress	“When you are sitting talking on the phone, then...Wolf is not...It is not as burdensome in that way, because you don’t have...You have the patients and patient contact, but you have it remotely. And in that way I find that my work environment has become easier, that we have days when you don’t have quite so much patient contact, and that is actually restorative. Having them remotely, to be able in some way...Even though it’s patient contact, it’s not direct patient contact, and that’s great for our recovery.” [nurse 2]	
	Flexibility	“It has gotten a little easier compared to otherwise, when I am at home. Then it is indeed...Then I have been able to do some of my job tasks from home rather than having to put them off until I am back in the workplace, so it has made things a little easier for me.” [psychologist 1]	
	Workarounds	“...we are accustomed to not being [laughs] synchronised with all the systems there are, but what happens is that we have to cope with this in a similar manner as we have done with certain systems before. So it’s not actually a surprise to us, but it is clear that it adds an extra work step.” [care manager]	
**Deviation from NPT constructs**			
	Materiality	“Yes, I receive more data. Because in Wolf it is the patients who determine completely what they want to reveal...or what you have on this questionnaire that I assume they are given for various symptoms they have, that’s what I think. And then it’s guided by the questions you have, so to speak, while with telephone contact I am the one who guides the actual questioning and zeroes in on the problem as quickly as I can, naturally.” [nurse 3]	

When asked about the expected benefits of using the digital platform, the interviewees commonly mentioned the possibilities of increased patient access, easier contact with and access to patients, less time spent on phone calls, and nurses spending less time trying to reach physicians for advice about patients and their symptoms. The physicians particularly highlighted the expected benefits for nurses: spending less time on the phone with patients, tools for triage, and increased flexibility. The nurses, on the other hand, particularly highlighted the expected benefits for patients—spending less time trying to reach the health care center by phone and having the option of contacting it through the digital platform. The interviewees also had very similar conceptualizations of how the digital platform and its utility differed from existing practices. NPT emphasizes that differentiation is part of the sense-making process, as it relates to understanding how an innovation and its use will produce a new practice and how the new practice differs from the existing practice [15]. The idea of new practices resulting from the use of the digital platform was welcomed by the interviewees. They perceived that the platform would enable them to *relocate* some of the work to patients, who would fill in their own symptoms and medical history, answer the follow-up questions provided automatically based on what the patients had filled in, and upload pictures. The envisaged benefits of using the digital platform include the digital platform collecting and providing detailed information about patients, thus providing a foundation for analysis and decision-making. This, together with the chat and video functionality, was seen as more efficient than current practices in which prospective patients generally contact the Albra primary care center by phone.

Although the value and benefits of using the digital platform were a positive fit with the interviewees’ worldview and mindset, it is clear from their responses that they perceived the platform as a supplement to the current practice, not as a replacement for it. Many commented that the digital platform provided new communication possibilities that suited some patients but not all.

### Cognitive Participation: Relationship Work That HCPs Do to Build and Sustain a New Practice

It is not enough that people understand and have a coherent view of a new practice. Practice will not change until people take personal responsibility and are committed to implementation. May et al [15] referred to this second construct of NPT as cognitive participation. It concerns individual and shared commitments and engagements in innovation and its implementation [15]. We identified four themes that were prominent for individual and mutual engagement and commitment in the implementation: *close collaboration*, *implementation champions*, *patient engagement,* and *familiarity*, as shown in Table 1.

It is clear from the data that the digital platform was implemented by the management in close collaboration with the HCPs employed at the health care center. The decision to implement it was taken by the management in close collaboration with one of the physicians and some of the nurses, who were all later trained to become *implementation champions*; that is, persons who drive the implementation and assist colleagues when they need help or encounter problems with the platform) [29]. Furthermore, the platform and its utility in practice were regularly discussed and scrutinized, both before and during the implementation, during formal meetings between the management and the employees, and during informal meetings between colleagues. Frequent meetings fostered commitment and engagement in the implementation. Moreover, the implementation was conducted in *close collaboration* with the platform provider. Initially, the platform provider presented a digital platform to the management and a small group of employees. Thereafter, the provider conducted half-day training sessions with the HCPs. Hence, implementation was not left to the management of the primary health care center or the HCPs themselves; instead, it was approached as a joint venture with the platform provider.

Interviewees commonly said that patient engagement with the platform was crucial. According to NPT, legitimation (ie, beliefs and ideas about whether HCPs feel that it is right for them to be involved and whether the new practice corresponds to their core values) is an important mechanism of cognitive participation. Another factor that affected the interviewees’ engagement in the use of the platform was familiarity. The *familiarity* of the user interface, as well as prior experience with chat and videoconferencing, affirmed commitment to the implementation as the interviewees had encountered synchronous (video calls) and asynchronous (chat and photo uploads) chats before. The interface and functionalities were thus perceived as familiar and easy to use.

The familiarity of the functionalities, as well as the willingness of HCPs and patients to use the platform, appear to have acted as facilitators for cognitive participation. The interviewees, both individually and together, engaged in the implementation by taking part in the discourse on the positive effects of the digitalization of health care and in feedback loops regarding the platform, which were scheduled at regular meetings. The act of articulating and rearticulating the benefits of using the platform seemed to be an important source of cognitive participation and a possible contributor to creating and maintaining commitment and engagement in implementation.

### Collective Action (Enacting Work): Operational Work That People Do to Enact a New Practice

According to NPT, collective action is the third construct of NPT [15]. Collective action refers to how a practice operationalizes (or does not) an innovation. It includes the actual work people do together and in relation to the implementation and the confidence HCPs have as individuals and as a team in the new practice. It also includes how work is outlined and allocated and how innovation is incorporated into institutional and social structures. The dominant themes related to collective action were *parallel work practices* and *new communication patterns* (Table 1).

When asked about how the digital platform was enacted in practice, the interviewees commonly said that there was time scheduled for nurses and physicians to work on the platform. During scheduled *platform time*, both nurses and physicians worked exclusively in the platform and did not take phone calls or meet patients in person. Thus, using the digital platform became a parallel new work routine alongside the *existing routines*; it became the supplement and enhancement to current practice mentioned during the sense-making work. In most cases, it was perceived as a change for the better. It enabled variation in work tasks and provided a source of respite while meeting the needs of patients at the same time.

Although the interviewees saw the benefits of the new working routines, some also highlighted the disadvantage of parallel work practices as the digital platform added to the complexity of work tasks. However, concerns about the additional complexity because of having one more system to attend to did not seem to have a major negative effect on the use of the platform. The data revealed that the interviewees felt confident in using the platform and in enacting new routines. They felt that they had received proper training and that they could receive help, if needed, from colleagues (implementation champions). Owing to the digital platform, they developed new patterns of communication, both between colleagues and with patients.

The interviewees felt that communication had become more efficient not only between different kinds of HCPs but also with patients. Some patient requests could now be handled through the digital platform, and automated triage, chat, video, and photo uploads enabled HCPs to help patients without having to book physical visits. The ability to use written communication challenged the HCPs’ habits, and they needed to develop or fine-tune their skills. This was a work in progress at the time of the interviews. Nevertheless, the interviews revealed that the platform had become embedded in everyday practice and ran in synergy with old routines. The different HCPs worked together with and through the platform. The transition from old routines to new routines using the platform was well-supported by the management, as the employees had scheduled time to use the platform. The HCPs had the same responsibilities as before (eg, the patient’s initial contact was with a nurse, who decided whether he or she could attend to the patient’s request or whether it should be forwarded to other HCPs). However, they perceived the new system as allowing more efficient communication with colleagues and patients.

### Reflexive Monitoring: Appraisal Work HCPs Do to Evaluate and Understand How a New Practice Affects Them and Others

Reflexive monitoring refers to continuous judgments and beliefs regarding innovation and new practices, its impact on collaboration within the health care center, and its impact on service quality and value for individual HCPs and management. Reflexive monitoring also includes whether and how individuals alter or suggest changes to a digital platform to enhance its usefulness [15]. The dominant themes identified in the data that related to reflexive monitoring were *ease of use*, *enhancing teamwork*, *relief and less distress*, *flexibility,* and *workarounds* (Table 1).

Reflexive monitoring is an ongoing process of continuous interaction with other NPT constructs. It is not the *last stage* of the implementation but a continual process that reinforces sense making, commitment, and engagement, as well as how the implementation is operationalized. During the time we conducted the study, the interviewees had several positive appraisals of the digital platform and its effect on their working environment. A prominent theme was the *ease of use.* They did not need to invest much time to understand how to use the platform, which was perceived as easy to use with an intuitive interface. The interviewees not only perceived the platform as *easy to use* and understand but also praised it for enhancing teamwork. Several of them mentioned that the platform enabled and facilitated teamwork between different HCPs. The platform did not *configure* them or negatively alter professional relations with patients or other HCPs; it supported their professional work, social roles, and responsibilities. The narrative of *relief and less stress* was pervasive in nurses’ accounts of using the platform.

Another positive appraisal regarding the platform was that it allowed *flexibility*. It facilitates accessibility without geographical boundaries (eg, HCPs can work from home, and patients can *see* HCPs through video calls without physically visiting the health care center). This type of accessibility was important as the implementation was initiated early in the COVID-19 pandemic. The platform was also perceived as enabling flexibility in terms of which patients were requested to handle and when. Nurses could go through the list of patient requests and decide which ones needed urgent attention; which they could handle themselves; and which needed to be forwarded to a physician, psychologist, or other HCP. Another aspect regarding flexibility was that the HCPs did not have to get hold of the patients but could communicate via the platform at any time, whereas the patient could read the message at a time convenient for them. Thus, the platform extended accessibility across time and space.

Although the general view of the platform was positive, the interviewees also emphasized that the new routines required some *workarounds*. For example, the digital platform was not compatible with patients’ electronic health care records (EHRs). As a result, the nurses used two screens when working with the digital platform: 1 for the EHR and 1 for the digital platform. They needed to be able to see the patient’s medical history in the patient health care record and compare it with the data in the digital platform; they also needed to copy and paste data from the digital platform into the patient health care records. This, in turn, raised questions about how much data could just be copied and pasted from the digital platform or whether summaries were needed of the data that patients contributed by answering automated questions and filling in forms. At the time of our study, most of the interviewees said that they simply copied and pasted data from the digital platform directly into the EHRs. They noted that this approach resulted in the scattering of patient data within patient health care records. The alternative to copying and pasting would be to extract the data collected in the digital platform; however, this would require more work and thus reduce efficiency. However, the copy-and-paste procedure may result in extra time having to be spent comprehending the data in the EHRs and thus may have a negative effect on patient safety in the long run. This was not something that the interviewees mentioned; instead, the workarounds were discussed during frequent feedback loops with colleagues and the management. All were under the impression that this might be solved in the future or was something they would have to adjust to.

### Materiality

The materiality (ie, the characteristics) of the platform and how it shaped and was shaped by the implementation cut across all NPT constructs. In our analysis, we decided to split the *materiality theme* from the NPT constructs as our understanding of NPT is that it gives ontological priority to humans and social structures (focusing on social mechanisms such as coherence, cognitive participation, enactment, and reflexive monitoring), whereas the materiality of the technology and the role and significance of the technology in the implementation are somewhat neglected. May et al [17] described the implementation process as follows:

Whenever some new way of thinking, acting, or organising is introduced into a social system of any kind, it is formed as a complex bundle—or better, an “ensemble”—of material and cognitive practices.

As described by May et al [17], the social and the material are intertwined during the implementation, and it is hard to untangle the bundle. In our analysis, the materiality of the platform did not fit seamlessly into any of the constructs of NPT. Verbeek [30] argues that human-technology relations expand beyond mere functionality and use. To understand how humans and technology coshape experiences and practices, one has to think in terms of hybrids [30]. Here, *hybrid* refers to the way human experience, perception, and practices result from elements of technology and contexts (ie, technology mediates human experience and practices). In our study, the HCPs communicated with each other and with patients through the digital platform, which mediated a different kind of communication (written instead of oral and asynchronous instead of synchronous). The platform was also used to collect data from the patient’s input, asking automated follow-up questions based on the patient’s answers. Consequently, the platform directed what kind of questions the patient was given, resulting in automated triage. Thus, the HCPs’ knowledge of the patients and what kind of care they need was mediated through the platform and its algorithms.

The materiality of the platform also affected how patients contacted the primary health care center. They were free to choose between different kinds of technologies. According to the interviewees, patients used different logic to achieve different goals; for example, patients with sexually transmitted diseases or mental illnesses preferred to seek help through the platform rather than by phone. Some patients made contact both by phone and via the digital platform, as the materiality of the technologies in practice enabled it. This, in turn, highlights that materiality is closely connected to usability and how design features are understood and used. However, Orlikowski [31] has shown in her work that the material and the social cannot be viewed separately as organizations, practices, people, social relations, power relations, digital systems, and so on and are interconnected [31]. In other words, materiality goes beyond focusing on material properties such as design features and interfaces. Furthermore, material properties are not stable entities but are subject to interpretations, reinterpretations, negotiations, and local adaptions [32]. Orlikowski [33] insists that it does not matter if a technology can do *X, Y, or Z*; if people do not perceive it as capable of doing *X, Y, or Z*, they will not use it, and as a result, the technology will not change the way they work [33].

What is interesting is that although the materiality of the digital platform supported certain actions while constraining others, the materiality of the platform and its mediating effect on practice was not questioned by the interviewees, as technological determinism reverberated in their accounts.

## Discussion

### Principal Findings

We used the constructs of NPT to make sense of our data and generalize the findings. The NPT constructs were visible in the data, and the theory helped us understand the important mechanisms involved while embedding and integrating the digital platform into everyday practice. NPT sheds light on the mechanisms and dynamics of the implementation process in a way that probably would not have been possible with frameworks such as the diffusion of innovations theory and the technology acceptance model or its derivatives [18,20,34]. Our findings indicate that the platform became normalized into everyday primary care routines. The work done before and during the implementation involved several actors and actions. The platform developers, who were clinicians, developed a platform to solve problems they faced when managing patients in everyday care practice. The development of the platform was iterative and involved HCPs throughout the development process. Thus, the problem and potential solutions were anchored in health care practices and easily translated by HCPs. Furthermore, the problem of inefficient patient management that the platform was expected to solve is well-known, both to HCPs and in society. As a result, it was readily accepted by patients as well.

Throughout the implementation, the management involved their employees, the HCPs (1) by initial presentations on and discussion about the digital platform and its potential in practice; (2) after the decision was made to implement the platform, training sessions were run by the platform provider; (3) key individuals were trained as implementation champions; (4) the HCPs had scheduled hours when they could work uninterrupted using the platform; and (5) there was ongoing discussion and reflection on the platform at formal meetings.

The HCPs had an equally important role in embedding the platform into everyday practice: (1) they interpreted digital communication with patients as a meaningful addition to current practice; (2) some took on the role of implementation champions and fostered the involvement and engagement of their colleagues by supporting them in their use of the platform and by an informal discussion of the platform; (3) they enacted the platform in current practice and communicated through the platform with colleagues and patients; (4) they reinforced organizational and social norms and values by praising the system for enhancing teamwork, increasing patient accessibility, and being a source of respite; and (5) they found workarounds to fit the platform into practice.

We perceived NPT and its constructs as beneficial as it helped to deepen the analysis and move away from identifying barriers to and facilitators of understanding general means for supporting implementations. However, a problem we encountered with using the constructs of NPT was related to presenting the data. As the findings are presented sequentially, one can obtain the impression that the implementation is a clear-cut, linear process. However, it is an ongoing complex process, as pointed out by Nilsen [10]. The constructs of NPT are intertwined and interrelated, and the implementation is a continuous process that reinforces sense making, commitment and engagement, and operationalization and appraisal or configuration [15,17]. Translation studies show that translations are never completed but continuously negotiated and renegotiated. Thus, an initially successful implementation may turn into a failed or abandoned implementation [35]. As the study presented in this paper concerns only the first 6 months of the implementation, it is difficult to know whether the actors will sustain their involvement and engagement with the platform and whether the system will become a sustainable part of everyday care practice.

A perceived drawback with NPT, as we understand it, is that it gives ontological priority to humans and social structures while the materiality of the technology is relegated to the background. However, the materiality of technology should not be neglected as every digital health care innovation designs certain kinds of care and working conditions or at least certain ways of providing care [36]. In our study, the materiality of the platform enabled communication between HCPs and both patients and colleagues across time and space (asynchronous communication without geographical boundaries). The platform required patient input, and the patient was guided through automated questions and forms. As a result, the platform not only stores and transmits information but also transforms the patient data process by automated triaging. Consequently, the patient’s experiences of health and sickness are enacted and reproduced through the platform and its algorithms. As such, the platform has performativity and affects the HCPs’ understanding of the patient’s medical status and, in turn, the care given. Thus, the platform, the patients, the HCPs, and the social contexts they are part of are entangled and coconstitutive of each other [31,37]. In other words, the platform and its materiality (eg, which actions and decisions it enables vs constrains) affect knowledge and power dynamics in primary care and, as a result, shape and mediate relations among HCPs, patients, and the health care center. This, in turn, raises ethical questions such as which norms and values are embedded in the platform and its algorithms. Further research is needed on the ethical dimensions of the platform and the effects of the platform’s performativity.

### Limitations of the Study

It is difficult to generalize about digital platform implementations in primary care and their effects on HCPs’ working conditions from a small-scale exploratory study such as this. However, as the constructs of NPT were used to make sense of our data, we identified reasons why the digital platform became embedded and integrated into the practice we studied and what health care organizations can do to support the implementation of digital health care platforms. Long-term research at multiple sites may be valuable to test and validate our findings, as well as to identify mechanisms promoting or inhibiting sustainable implementation.

As the data collection and analysis were guided by the theoretical framework of NPT, we may have been blinded by the theory and thus failed to identify aspects of implementation that are not described by NPT. In an attempt to avoid this, we first analyzed the data using an inductive approach, searching for themes emerging from the data. After that, we deductively tested whether the NPT constructs were visible in the data.

### Conclusions

Changing primary care practices from patient consultations by phone and physical visits to digital communication and automated patient assessments allows for increased patient accessibility across time and space. At the same time, digital solutions in primary care challenge current care practices and affect the working routines of HCPs. In this paper, we have shown how a digital health care platform became embedded and integrated into care practice as the HCPs perceived it as beneficial. They were also actively involved in the implementation, had scheduled hours for working with patients through the platform, and had ongoing support from management and implementation champions. Furthermore, the platform and its materiality (eg, which actions and decisions it enables vs constrains) shaped and mediated relations among HCPs, patients, and the health care center. The findings imply that implementation outcomes for changing work practices do not rely only on microlevel actions and engagements, as shown by the NPT analysis. Implementations also depend on interrelations with macrolevel conditions such as the current COVID-19 pandemic (eg, physical distancing) and the dominant discourse on digitalization as a prerequisite for efficiency and accessibility. This, in turn, circles back and influences microlevel actions and engagements.

## Abbreviations

EHR: electronic health care record
HCP: health care professional
NPT: normalization process theory
